# Intranasal H5N1 Vaccines, Adjuvanted with Chitosan Derivatives, Protect Ferrets against Highly Pathogenic Influenza Intranasal and Intratracheal Challenge

**DOI:** 10.1371/journal.pone.0093761

**Published:** 2014-05-21

**Authors:** Alex J. Mann, Nicolas Noulin, Andrew Catchpole, Koert J. Stittelaar, Leon de Waal, Edwin J. B. Veldhuis Kroeze, Michael Hinchcliffe, Alan Smith, Emanuele Montomoli, Simona Piccirella, Albert D. M. E. Osterhaus, Alastair Knight, John S. Oxford, Giulia Lapini, Rebecca Cox, Rob Lambkin-Williams

**Affiliations:** 1 Retroscreen Virology, London, United Kingdom; 2 Viroclinics Biosciences BV, Rotterdam, Netherlands; 3 Archimedes Development Limited, Nottingham, United Kingdom; 4 University of Siena, Siena, Italy; 5 VisMederi LifeSciences, srl, Siena, Italy; 6 Department of Viroscience, Erasmus MC, Rotterdam, Netherlands; 7 Evicom, Teddington, United Kingdom; 8 Department of Clinical Science, University of Bergen, Bergen, Norway; 9 Department of Research and Development, Haukeland University Hospital, Bergen, Norway; Mount Sinai School of Medicine, United States of America

## Abstract

We investigated the protective efficacy of two intranasal chitosan (CSN and TM-CSN) adjuvanted H5N1 Influenza vaccines against highly pathogenic avian Influenza (HPAI) intratracheal and intranasal challenge in a ferret model.

Six groups of 6 ferrets were intranasally vaccinated twice, 21 days apart, with either placebo, antigen alone, CSN adjuvanted antigen, or TM-CSN adjuvanted antigen. Homologous and intra-subtypic antibody cross-reacting responses were assessed. Ferrets were inoculated intratracheally (all treatments) or intranasally (CSN adjuvanted and placebo treatments only) with clade 1 HPAI A/Vietnam/1194/2004 (H5N1) virus 28 days after the second vaccination and subsequently monitored for morbidity and mortality outcomes. Clinical signs were assessed and nasal as well as throat swabs were taken daily for virology. Samples of lung tissue, nasal turbinates, brain, and olfactory bulb were analysed for the presence of virus and examined for histolopathological findings.

In contrast to animals vaccinated with antigen alone, the CSN and TM-CSN adjuvanted vaccines induced high levels of antibodies, protected ferrets from death, reduced viral replication and abrogated disease after intratracheal challenge, and in the case of CSN after intranasal challenge. In particular, the TM-CSN adjuvanted vaccine was highly effective at eliciting protective immunity from intratracheal challenge; serologically, protective titres were demonstrable after one vaccination. The 2-dose schedule with TM-CSN vaccine also induced cross-reactive antibodies to clade 2.1 and 2.2 H5N1 viruses. Furthermore ferrets immunised with TM-CSN had no detectable virus in the respiratory tract or brain, whereas there were signs of virus in the throat and lungs, albeit at significantly reduced levels, in CSN vaccinated animals.

This study demonstrated for the first time that CSN and in particular TM-CSN adjuvanted intranasal vaccines have the potential to protect against significant mortality and morbidity arising from infection with HPAI H5N1 virus.

## Introduction

Avian Influenza (H5N1) continues to present a significant risk to human health [1],[2],[3],[4], and recent genetic studies of H5 Hemagglutinin (HA) in an H1N1 virus backbone identified only four mutations in the HA protein were required to facilitate transmission in the ferret model emphasizing this threat [5]. Antigenic variations amongst H5N1 subtypes alongside the poor immunogenicity of the HA have both presented vaccine developers with difficulties [6], [7]. Influenza viruses undergo constant evolution via antigenic drift, and thus considerable antigenic and genetic diversity exists among currently circulating H5N1 viruses.

Most H5N1 vaccines that have demonstrated high immunogenicity required co-administration of an adjuvant and administration by the intramuscular route [8], [9], [10]. According to published literature, various adjuvanted vaccines have been shown to be able to reduce mortality in ferret challenge models but have not a) induced 100% seroconversion, or b) completely prevented virus replication in the respiratory tract. While protection from death is the most critical attribute for a pandemic vaccine, preventing viral shedding in the respiratory tract is a crucial additional step to interrupt population level transmission in a well vaccinated population (at least 60% coverage of healthy individuals for epidemic vaccination) [4],[11].

A ferret model was used in this study as ferrets resemble disease in humans when infected with Influenza A viruses [12],[13]. This ferret model is also the gold standard to demonstrate both the immunogenicity and the protective efficacy of Influenza vaccines [14],[15],[16],[17],[18].

Chitosan and its derivatives have been widely investigated as adjuvants for mucosal vaccination and for the intranasal delivery in particular [19]. Chitosan is a co-polymer of D-glucosamine and N-acetyl-D-glucosamine in which the amino groups provide a positive charge in aqueous solution. Chitosan is available commercially in water-soluble salt forms, such as glutamate and hydrochloride. Although chitosan salts are largely insoluble above about pH 6, a number of derivatives with enhanced solubility at neutral pH are available. One such derivative is trimethyl chitosan, in which some primary amine groups are replaced with methyl groups to provide increased solubility in neutral and basic environments [20].

The purpose of this study, in the ferret viral challenge model, was to investigate the protective efficacy of ChiSys (Archimedes Development Limited), a chitosan-based bioadhesive mucosal delivery system, as an intranasal adjuvant for an inactivated subunit vaccine containing modified HA and NA (Neuraminidase) antigens from A/Vietnam/1194/2004 (H5N1). A glutamate salt form (CSN) and a trimethyl derivative of chitosan (TM-CSN) were evaluated separately as adjuvants.

## Materials and Methods

### Viruses and virus reagents

The inactivated Influenza subunit vaccine was prepared from NIBRG-14, a vaccine seed strain that is a reassortant between PR8 and A/Vietnam/1194/2004 (Batch No. 1090/10)] that was kindly supplied by Novartis Vaccines and Diagnostics S.r.l., Italy. The ferrets were challenged with wild-type Influenza A/Vietnam/1194/2004 [H5N1] virus. The challenge virus was isolated from the field and passaged twice in Madin-Darby canine kidney cells (MDCK, ATCC-CCL-34).

Prior to the study, all ferrets were screened (using the HAI assay) against the following virus vaccine seed strains (reassortants that bear the Influenza PRB backbone but retain the HA and NA from the wild type viruses) were used in the assay: A/Vietnam/1194/2004 NIBRG-14 [H5N1] with the modified HA, A/Victoria/210/2009 [H3N2] (NYMCx187), A/California/7/2009 [H1N1] (NYMCx181). In addition to the above vaccine seed strains, the wild type virus B/Brisbane/60/2008 was used in the screen. All of these viruses were supplied by NIBSC, UK.

The vaccine responses in serum were measured with the HAI assay (Haemagglutination inhibition) by Viroclinics using the NIBRG-14 virus provided by NIBSC, UK. For HAI and Single Radial Haemolysis (SRH) assays performed by VisMederi srl., the viral antigens used were A/Vietnam/1194/2004 [H5N1] as supplied by NIBSC, A/turkey/Turkey/1/2005 [H5N1] as supplied by NIBSC, and A/Indonesia/5/2005 [H5N1] as supplied by CBER. For the viral neutralization assays (VN) the live viruses used were: A/Vietnam/1194/2004 [H5N1] as supplied by NIBSC, A/turkey/Turkey/1/2005 [H5N1] as supplied by NIBSC, and A/Indonesia/5/2005 [H5N1] as supplied by the CDC.

### Ferrets & Study Procedures

Thirty-six healthy outbred male ferrets approximately 12 months of age, between 1350 g and 2575 g in weight, were purchased from a commercial breeder. Animals were housed and experiments were conducted in compliance with EU directive 86/609/EEC) and Dutch legislation (Experiments on Animals Act, 1997). The protocol was approved by the independent animal experimentation ethical review committee of the Netherlands Vaccine Institute (permit number 201100332). Additional information on animal husbandry can be found in the File S1.

All ferrets tested negative for the presence of HAI antibodies against the challenge virus and recent seasonal strains of Influenza and Aleutian disease virus.

Three days prior to the first immunisation, the animals had temperature transponders implanted into the peritoneal cavity (DST micro-T ultra-small temperature logger; Star-Oddi, Reykjavik, Iceland). This device recorded the body temperature of the animals every 10 minutes. Effect of virus infection on body temperature was assessed for changes in the temperature of each ferret post inoculation. Body weights were measured at 1 and 7 days before inoculation and on 1, 2, 3, 4 and 5 days post inoculation (dpi). Serum was taken days 0, 21, 42, and 49 for hemagglutination inhibition (HAI), virus neutralization (VN), and single radial haemolysis (SRH) serology assays. Nasal and throat swabs were taken on 1, 2, 3, 4 and 5 dpi.

### Vaccine formulations

The intranasal vaccine candidates evaluated in the study were aqueous solutions containing an inactivated NIBRG-14 H5N1 subunit antigen, adjuvanted with ChiSys (Archimedes Development Limited, UK) utilising either chitosan glutamate (CSN) or N,N,N-trimethylated chitosan (TM-CSN) as in Table 1. As controls, antigen alone (unadjuvanted vaccine) chitosan-free vaccine formulation and placebo [phosphate buffered saline (PBS)] treatments were also tested.

**Table 1:** Summary of ferret treatment groups and vaccine and adjuvant formulations tested.

Group	Animals per group	Treatment description	Formulation details (total HA dose)	Route of vaccine administration	Days of Immunisations	Route of virus challenge
1	6	CSN Adjuvanted	0.075 mg/ml HA+5 mg/ml CSN (15 µg)	Intranasal	0, 21	IN
2	6	CSN Adjuvanted	0.075 mg/ml HA+5 mg/ml CSN (15 µg)	Intranasal	0, 21	IT
3	6	Unadjuvanted/Antigen alone	0.075 mg/ml HA (15 µg)	Intranasal	0, 21	IT
4	6	TM-CSN Adjuvanted	0.075 mg/ml HA+5 mg/ml TM-CSN (15 µg)	Intranasal	0, 21	IT
5	6	Placebo	PBS	Intranasal	0, 21	IN
6	6	Placebo	PBS	Intranasal	0, 21	IT

All materials for dosing to ferrets were supplied by Archimedes. The inactivated Influenza subunit vaccine HA content was measured using the SRID assay (Single Radial Immunodiffusion). Stock material was diluted with PBS or the appropriate CSN/TM-CSN adjuvant prior to use. The CSN adjuvant utilised chitosan glutamate 75–90% deacetylated, obtained from FMC BioPolymer AS, Norway. Stock CSN solutions were prepared in PBS. TM-CSN adjuvant was 77.7% deacetylated obtained from KitoZyme, Belgium. Stock TM-CSN solutions were prepared in ultrapure water. PBS tablets, Sigma, UK were used to prepare 0.01 M PBS, pH 7.4 in ultrapure water as per the manufacturer's instructions.

### Intranasal Immunisation

Animals were separated into six groups of six ferrets. Animals were immunised intranasally with 200 µl of the appropriate treatment divided between both nostrils, according to treatment assignments in Table 1, on Days 0 and 21 using a positive displacement automatic pipette with filter tip. As illustrated in Table 1 the treatment groups consisted of CSN adjuvant +15 µg/dose (n = 12), TM-CSN +15 µg/dose (n = 6), unadjuvanted +15 µg/dose (n = 6), and Placebo-PBS (n = 12). The CSN and Placebo groups were split into two and challenged by either the intranasal or intratracheal routes, while the TM-CSN and unadjuvanted treatment groups were only challenged by the intratracheal route.

### Inoculation with homologous H5N1 Influenza: A/Vietnam/1194/2004 (clade 1)

Four weeks after the last immunisation (day 49), all ferrets were challenged with wild-type Influenza A/Vietnam/1194/2004 [H5N1] virus. The challenge stock (7.3 log_10_TCID_50_/mL) was diluted in ice-cold PBS to target nominal concentrations of 3.3×10^4^ TCID_50_/mL for intratracheal challenge and 3.3×10^5^ TCID_50_/mL for intranasal challenge. The respective virus concentrations were selected in order to administer a nominal viral dose of 10^5^ TCID_50_ to each animal in a 3 mL volume by the intratracheal route and the 0.3 mL intranasally. After dilution, the challenge virus was kept on wet ice throughout its usage. Preparation and administration of the challenge virus inoculums were performed under BSL3+ conditions. A sample of each challenge virus dilution was titrated on MDCK cells (ATCC-CCL-34), as previously described [21]; back titration confirmed that all ferrets received a total HPAI virus inoculum of 7.5×10^4^ TCID_50_.

### Specimens for Viral Analysis

Nasal swabs and throat swabs for viral assays were collected daily into cold viral stabilisation medium (EMEM containing bovine serum albumin (fraction V), penicillin, streptomycin, amphothericin-B, L-glutamine, sodium bicarbonate and Hepes), aliquoted and stored at −70°C as previously described [21].

After necropsy, cranioventral, craniodorsal, caudoventral and caudodorsal sections of all lobes of the right lung and the accessory lobe (pooled per animal), right nasal turbinate and sections of the right brain and right olfactory bulb from each animal were collected for quantification of Influenza. All samples were stored at −70°C prior to further processing.

### Viral Quantification Assays

Infectious titres were determined by virus titration on MDCK cells, as previously described [21]. Influenza viral load for nasal swabs and throat swabs for tissues culture were measured as log (base 10) tissue culture infectious dose units/mL (log TCID_50_/mL) with a lower limit of quantification (LLOQ) of 0.8 log_10_ TCID_50_/mL. Analysis of the turbinates, lung, and olfactory bulb by tissue culture were expressed as log_10_TCID_50_/gram of tissue. The LLOQ of the tissue samples varied according to the tissue weight.

### Serologic Testing

Serum samples collected prior to the first and second immunisations and prior to inoculation were stored at −20°C. They were tested against homologous virus as well as clade 2.1 (A/Indonesia/05/2005) and 2.2 (A/Turkey/Turkey/1/2005) H5N1 viruses using HAI (utilizing both turkey and horse erythroctyes), VN, and SRH assays.

Sera were analyzed for the presence of anti-HA antibodies against A/Vietnam/1194/2004 (performed at Viroclinics with an initial dilution 1∶5), A/Indonesia/05/2005 (performed at VisMederi srl, Siena Italy with an initial dilution 1∶10), and A/Turkey/Turkey/1/2005 (also performed at VisMederi srl) by using an HAI assay with 1% turkey erythrocytes, as described previously [22]. Additionally the modified HAI method from Stephenson et al, 2004 was used to measure H5N1 HAI responses in 1% horse erythrocytes by VisMederi srl (initial dilution of 1∶10). Neutralisation assays were performed at VisMederi srl, as described by Svindland *et al* [23] (additional information on the neutralization assay can be found in the File S1). MDCK-SIAT1 cells were obtained from ECACC ref. 05071502. The Spearman-Kärber formula was used to calculate the neutralisation titre of each sample [24], [25]. Ferrets that had a ≥4 fold rise in their HAI or VN titre were identified as seroconverted. Any of Days 21, 42, and 48 could be used when defining seroconversion. Single radial haemolysis was performed at the University of Siena, Italy, based on the modified reference method standardised by Schild *et al* [26] and performed as described in detail by Svindland *et al* [23]. A haemolysis area <4 mm^2^ was considered negative, between 4 and 25 mm^2^ positive but not protective, and greater than 25 mm^2^ as positive seroprotective, as per European Medicines Agency & the Committee for Medicinal Products for Human Use (EMEA CHMP) guidelines [27]. Positive control serum sample (sheep hyperimmune sera) was supplied by the National Institute for Biological Standards and Control, UK).

### Pathology

At the time of necropsy, a complete macroscopic post-mortem examination was performed, the animals were weighed and abnormalities were recorded. All lung lobes were inspected and lesions described. The lungs were collected and weighed. The relative lung weight was calculated as proportion of the body weight (lung weight/body weight at necroscopy×100). The right lung was sampled for virology. The left lung and left nasal turbinates were fixed with 10% neutral buffered formalin for histopathology. After fixation, sections from the cranial- and caudal lobes (n = 2 each) and nasal turbinates were embedded in paraffin and the tissues sections stained with haematoxylin and eosin and histopathological examination performed as described elsewhere [28].

### Statistical Analysis

Statistical analyses and construction of figures were performed with GraphPad Prism Software v5.0 (La Jolla, CA). ANOVA and Fisher's exact test analyses were two tailed, statistical significance was set at p = 0.05. Additional information on specific analysis can be found in the File S1.

## Results

Ferrets were observed after each of the vaccinations for general health. There was no untoward vaccine post-administration observation noted for the four intranasal treatments.

### Vaccination of Ferrets with H5N1 Vaccines and Serologic Responses

**Figure 1:**
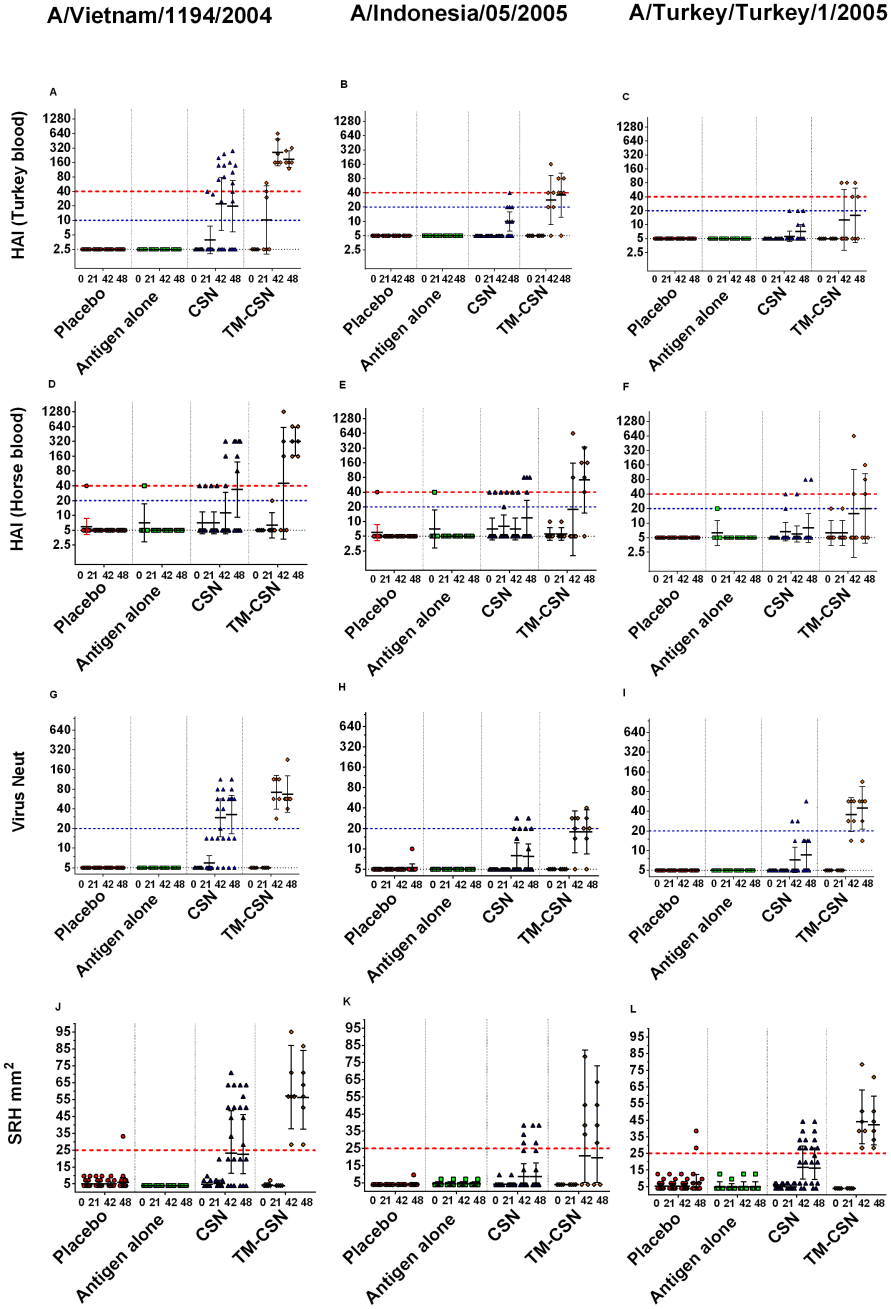
Antibody responses of immunised ferrets to A/Vietnam/1194/2004 (clade 1), A/Indonesia/05/2005 (clade 2.1), & A/Turkey/Turkey/1/2005 (clade 2.2) H5N1 viruses by HAI (turkey and horse erythrocytes), VN, and SRH assays. All animals were intranasally immunised on days 0 and 21. Data presented for each of the vaccine groups before and after 1 vaccination (Day 0 and Day 21), 2 vaccinations (Day 42), and just prior to challenge (Day 48). Treatment groups were Placebo (PBS) n = 12; unadjuvanted/antigen alone (0.075 mg/mL HA) n = 6; CSN adjuvanted vaccine (0.075 mg/mL HA+5 mg/mL CSN) n = 12; & TM-CSN adjuvanted vaccine (0.075 mg/mL HA+5 mg/mL CSN) n = 6. Bars represent geometric mean group values (horizontal bars) and ±SD (vertical bars). Vaccine responses are as follows: graphs A, D, G, & J are responses to A/Vietnam/1194/2004, as measured by HAI (turkey erythrocytes), HAI (horse erythrocytes), VN & SRH respectively; B, E, H, & K are responses to A/Indonesia/05/2005; C, F, I, & L are responses to A/Turkey/Turkey/1/2005. Seroconverting ferrets (threshold represented by blue horizontal dotted line) were defined as those animals with an equal or greater than 4 fold increase in titre from baseline for the HAI and VN assays. Those that attain an area of 25 mm^2^ or more for the SRH assay were defined as seroprotected. Seroprotection levels for the HAI assay were defined as equal or greater than 40HAI (threshold represented by red horizontal dotted line).

Sera from vaccinated ferrets were tested with HAI, SRH, and VN assays against the homologous challenge virus (clade 1) as well as representatives from clade 2.1 and clade 2.2 (Tables S1,S2,& S3 in File S1 respectively). None of the six ferrets vaccinated with antigen alone (15 µg HA/dose) showed significant serum antibody titres after two vaccinations by any assay against either homologous virus or antigenically distinct viruses (Figure 1, & Tables S1, S2, & S3 in File S1). After a single dose of CSN adjuvanted vaccine 2 out of 12 ferrets seroconverted by HAI (turkey erythrocytes) to homologous virus, and after two vaccinations 7 out of 12 seroconverted by the HAI (turkey erythrocytes) assay, 7 attained seroprotective levels in the SRH assay, with 8 out of 12 seroconverting as measured by the VN assay. Of those vaccinated with the TM-CSN adjuvant 3 out of 6 seroconverted by HAI (turkey erythrocytes) to homologous virus after one vaccination. After two vaccinations, 6 out of 6 seroconverted by HAI (turkey and horse erythrocytes) and VN assays, and all six attained seroprotective levels measured in the SRH assay.

We assessed cross-clade immunogenicity of the adjuvanted intranasal vaccines for clade 2.1 (Figures 1B, 1E, 1H, & 1K) and 2.2 (Figures 1C, 1F, 1I, & L) viruses.

In animals that received two CSN adjuvanted vaccinations 6 out of 12 animals attained seroprotective levels for A/Turkey/Turkey/1/2005 by SRH assay, with 2 out of 12 seroconverting by each of the other assays. Four, 2 and 3 out of the 12 animals seroconverted to A/Indonesia/05/2005 (clade 2.1) by HAI (turkey erythrocytes), HAI (horse erythrocytes), and VN assays respectively with 3 ferrets attaining seroprotective levels in the SRH assay.

In the TM-CSN adjuvant vaccinated ferrets 4 out of 6 ferrets attained seroprotective levels for A/Indonesia/05/2005 (by SRH assay). The other serological methods detected seroconversion in 5, 5 and 4 out of 6 animals by HAI (turkey erythrocytes), HAI (horse erythrocytes), and VN assays respectively. In the TM-CSN adjuvant vaccinated ferrets 6 out of 6 ferrets attained seroprotective levels for A/Turkey/Turkey/1/2005 (by SRH assay). The other serological methods detected seroconversion in 3, 2 and 6 out of 6 animals by HAI (turkey erythrocytes), HAI (horse erythrocytes), and VN assays respectively.

In addition to using turkey erythrocytes for the HAI assay for the 3 virus clades, we also used horse erythrocytes. Assays using horse erythrocytes mostly detected either an equivalent or a lower number of seroconverting ferrets than when using turkey erythrocytes. In two cases horse erythrocytes allowed for identifying seroconversion that was not picked up with turkey erythrocytes (CSN adjuvanted group after 1 vaccination against the clade 2.1 and 2.2). This difference is not explained by the difference in LOD and therefore the seroconversion threshold definition between the assays. Otherwise the horse blood results were similar in titre in cases when both assays detected HAI antibodies.

### Protective Efficacy of Intranasal Vaccination vs Homologous HPAI A/Vietnam/1194/2004 [H5N1] Virus, with either Lethal Intratracheal Inoculation or Intranasal Inoculation

#### Mortality

**Figure 2:**
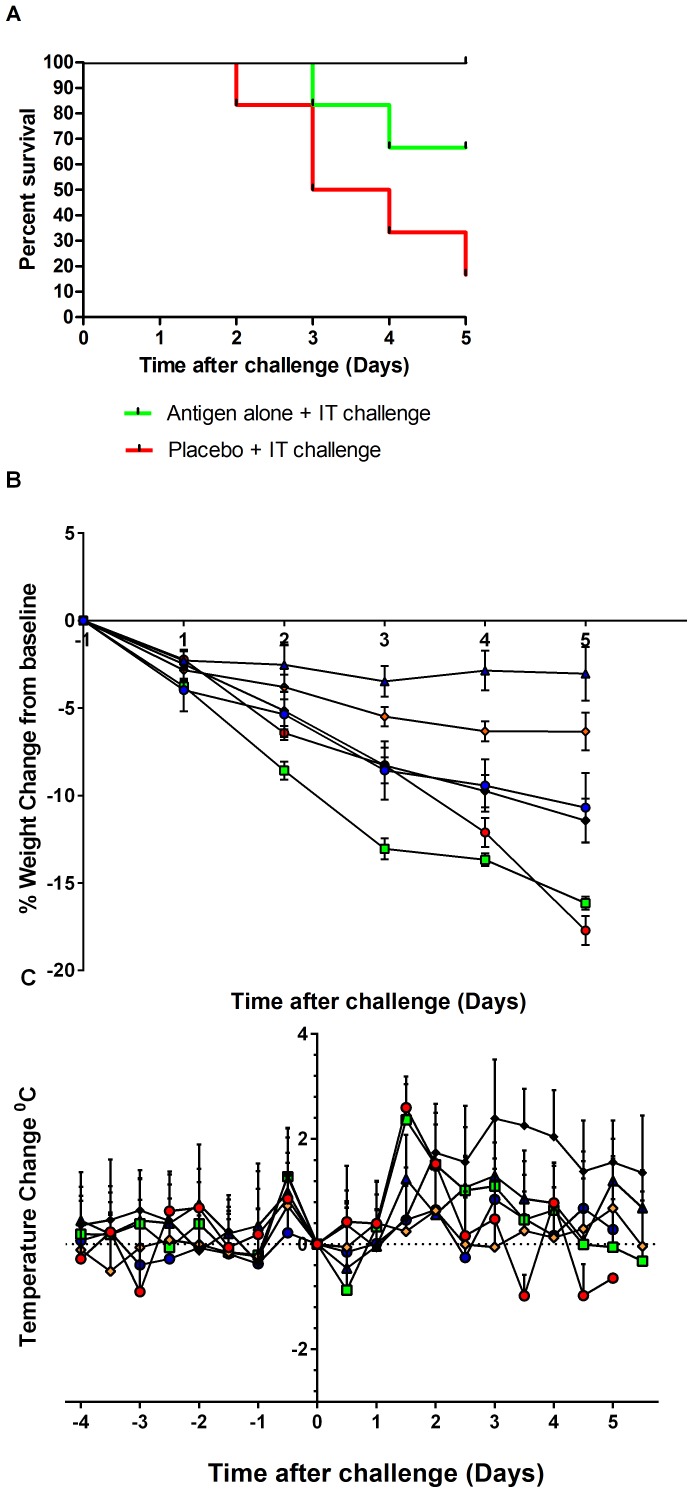
Morbidity and mortality in in control and vaccinated ferrets post challenge with homologous H5N1 HPAI virus. 2A. Kaplan Meyer survival plot for vaccinated ferrets post challenge. The following groups were challenged with HPAI virus by the intratracheal route and the data is shown in 2A: red line = placebo group challenged, green line = unadjuvanted/antigen alone vaccine group, all other groups are represented by the black line. 2B.daily percentage weight change post challenge. Treatment group mean weight changes (%) on each day post challenge are represented with ± SEM. 2C. Temperature change (°C) post challenge. Ferret temperature was monitored every 10 minutes with implantable thermometers allowing analysis of temperature change from baseline (average of Days 45–48 temperatures for each animal). Treatment group mean temperature changes every 12 hrs (00∶00 and 12∶00) are represented with ± SD. The following groups were challenged with HPAI virus by the intratracheal route and the data is shown in 2B and 2C: red circle = placebo group challenged, green squares = unadjuvanted/antigen alone vaccine group, blue pyramids = CSN adjuvanted vaccine, orange diamonds = TM-CSN adjuvanted vaccine. The following groups were challenge by the intranasal route: black diamonds = placebo, and blue circles = CSN adjuvanted vaccine.

Intranasal challenge of influenza naïve ferrets produced virus replication predominantly in the upper respiratory tract (URT) and central nervous system (CNS) and none of the six animals had to be euthanised on welfare grounds. In comparison, the intratracheal route of inoculation resulted in a predominantly lower respiratory tract infection (LRT) which was associated with a significantly higher mortality (p<0.05). Thus 5 out of 6 animals were either found dead or were euthanised prematurely (Figure 2A). High viral titres in the swabs and tissues, and marked histopathological changes and weight loss were noted in these animals.

Vaccination with antigen alone (15 µg HA/dose) did not protect 2 of 6 animals from premature death. In contrast both the CSN and TM-CSN adjuvanted vaccines protected all ferrets from the lethal outcome of intratracheal inoculation. In the groups inoculated intranasally, including placebo, all animals survived.

#### Body Weight loss & Body temperature change

*Placebo* animals inoculated intratracheally showed more consistent body weight loss compared to intranasally inoculated ferrets. The CSN vaccinated animals showed significantly reduced AUC in weight loss (p<0.05) when compared to both intratracheal and intranasally challenged placebo ferrets (Figure 2B). TM-CSN vaccinated animals had reduced AUC weight loss but this did not reach significance (p = 0.09). Both the intracheally inoculated placebo and antigen only groups exhibited a sharp rise in temperature a day after challenge that resolved the following day (Figure 2C). The intranasally inoculated placebo group had a high temperature from Day 2 that remained elevated until the end of the study. Vaccination with the CSN adjuvant and antigen reduced the mean peak temperature rise from baseline (not significant p = 0.066) compared to placebo when challenged by the intranasal route. In contrast when challenged by the intratracheal route of infection, both the CSN and TM-CSN vaccinated ferrets exhibited significantly reduced mean peak temperature change compared to the placebo group (p<0.05).

#### Viral load in the respiratory tract and CNS

Table 2 shows the number of ferrets in each of the vaccine groups that had culturable virus in either of the URT, LRT, or CNS.

**Table 2:** Correlation of serological response to vaccination and subsequent mortality, clinical signs, and virus detection in the respiratory tract and CNS.

	Seroconversion and seroprotection against A/Vietnam/1194/2004				Clinical Signs		Viral detection			
2×Intranasal Vaccination (15 µg)	HAI sero conversion	VN sero conversion	SRH sero protective	No. dead/total No.	Temperature increase °C	Weight change %	URTI	LRTI	CNS	ANY
Intratracheal challenge										
Unadjuvanted/Antigen alone	0/6	0/6	0/6	2/6	3.12	−14.82	6/6	6/6	4/6	6/6
CSN Adjuvanted	3/6	4/6	4/6	0/6 ^*^	2.37	−4.76	3/6	2/6	0/6^*^	3/6
TM-CSN Adjuvanted	6/6 ^*^	6/6 ^*^	6/6 ^*^	0/6 ^*^	2.03	−7.33	0/6^*^	0/6^*^	0/6^*^	0/6^*^
Placebo (PBS)	0/6	0/6	0/6	5/6	3.49	−12.04	6/6	6/6	5/6	6/6
Intranasal challenge										
CSN Adjuvanted	4/6	4/6	3/6	0/6	2.36	−11.09	6/6	0/6	2/6	6/6
Placebo (PBS)	0/6	0/6	0/6	0/6	2.94	−11.44	6/6	0/6	5/6	6/6

Values in bold and with an * - represent significant differences compared to placebo; p<0.05 by Fisher's exact test, two tailed.

HAI seroconverted proportion includes those ferrets that had a ≥4 fold rise from baseline/total number of ferrets.

VN seroconverted proportion includes those ferrets that had a ≥4 fold rise from baseline/total number of ferrets.

SRH seroprotected proportion include those ferrets that had a ≥25 mm^2^ haemolysis area/total number of ferrets.

URTI includes daily samples from nasal turbinates, nasal swabs, and throat swabs.

LRTI includes lung samples.

CNS includes brain and olfactory bulb samples.

ANY includes URTI, LRTI, or CNS.

There was no detectable virus in the TM-CSN vaccinated ferret samples from the URT, LRT, or CNS. In contrast, virus was isolated from 3 out of 6 ferrets in the URT (throat swabs), and 2 out of 6 ferrets in the LRT (lung sample) that received CSN adjuvanted vaccine following intratracheal challenge.

**Figure 3:**
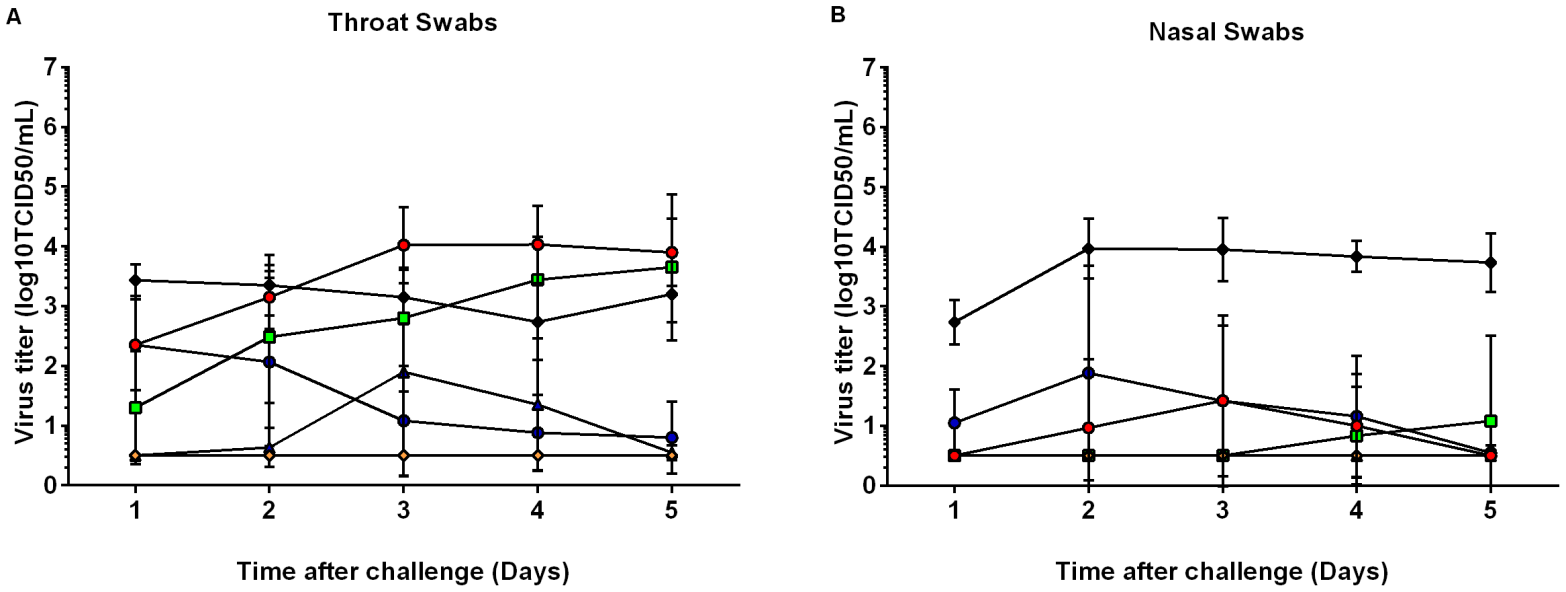
Virus Titres in Throat- and Nasal Swabs in control and vaccinated ferrets post challenge. Both throat swabs (3A) and nasal swabs (3B) were analysed for viral loads by cell culture (log_10_TCID_50_/mL) and plotted daily after challenge. Plots contain mean daily mean titres with ± SD for each treatment. The following treatment groups were challenged with HPAI virus by the intratracheal route: red circle = placebo group challenged, green squares = unadjuvanted/antigen alone vaccine group, blue triangles = CSN adjuvanted vaccine, orange diamonds = TM-CSN adjuvanted vaccine. The following groups were challenge by the intranasal route: black diamonds = placebo, and blue circles = CSN adjuvanted vaccine.

Intranasal CSN adjuvanted vaccinated ferrets had significantly lower mean AUC of nasal and throat swab titres (mean titres of each group by day are shown in Figure 3) than placebo (p<0.05), as well as significantly lower mean AUC of throat swab virus titres when challenged by the intratracheal route (p<0.05). Those ferrets that received TM-CSN vaccine and were challenged intratracheally had significantly lower mean AUC virus titre in throat swabs when compared to placebo (p<0.05).

**Figure 4:**
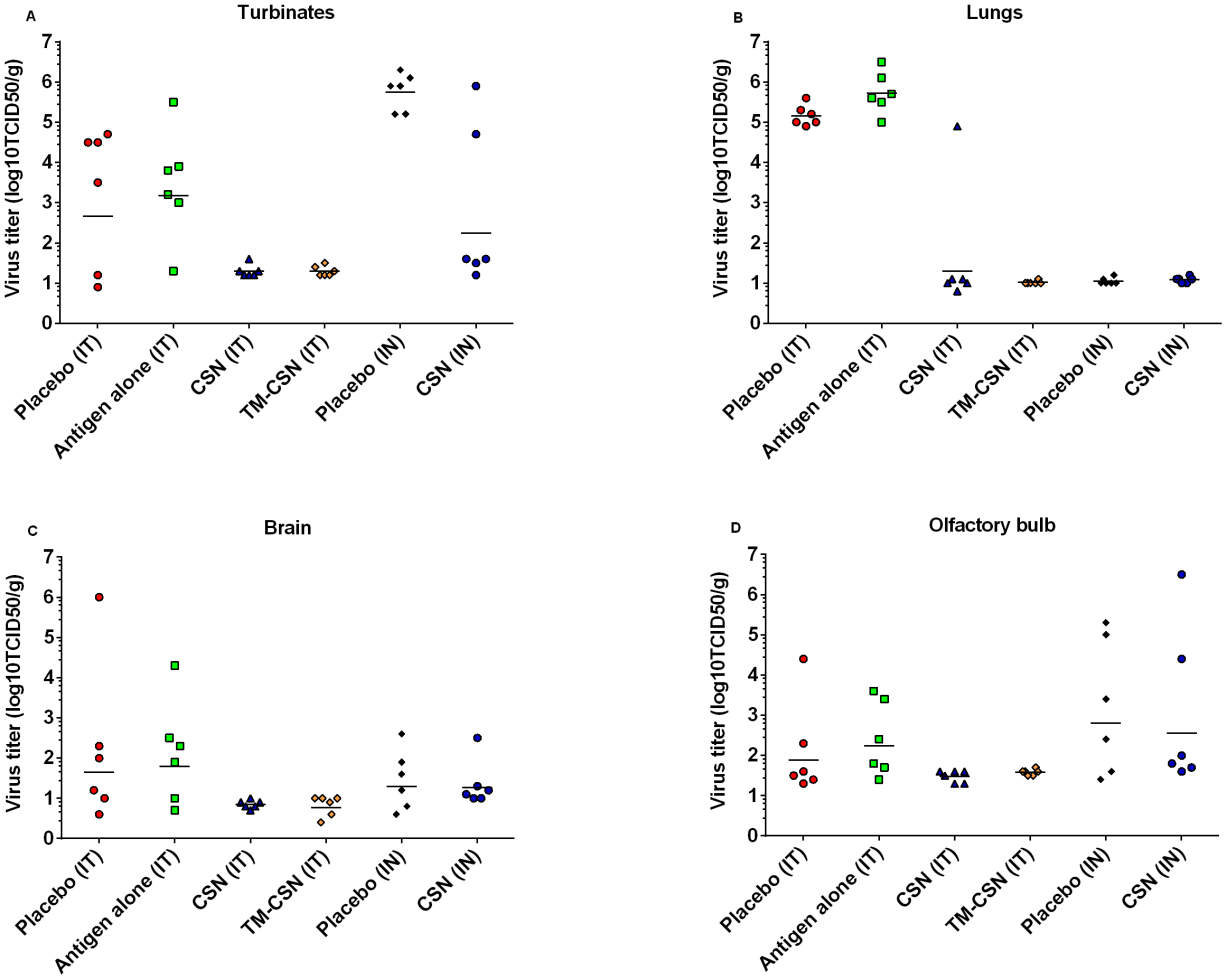
Virus Titres in Nasal Turbinates, Lungs, Brain, and Olfactory bulb in control and vaccinated ferrets post challenge. On the day that each ferret was euthanised samples taken from the turbinates (A), lung (B), brain (C), and olfactory bulb (D) were analysed for viral loads by cell culture (log_10_TCID50/gram) and plotted as a scatter plot with geometric mean titres for each group. The following groups were challenged with HPAI virus by the intratracheal route: red circle = placebo group challenged, green squares = unadjuvanted/antigen alone vaccine group, blue triangles = CSN adjuvanted vaccine, orange diamonds = TM-CSN adjuvanted vaccine. The following groups were challenge by the intranasal route: black diamonds = placebo, and blue circles = CSN adjuvanted vaccine.

The mean viral titres of lungs, turbinates, brain and the olfactory bulb at the day of death from TM-CSN and CSN treatment groups were all lower than placebo (Figure 4), when challenged by the intratracheal route. However it should be noted that possible bias from the timing of death in the ferrets cannot be discounted for comparisons involving the IT challenged placebo group and the antigen alone group for viral titres in the lungs, turbinates, brain and the olfactory bulb. CSN vaccinated animals had significantly lower mean nasal turbinate virus titres than placebo when challenge by the intranasal route (p<0.05).

#### Histopathology

**Figure 5:**
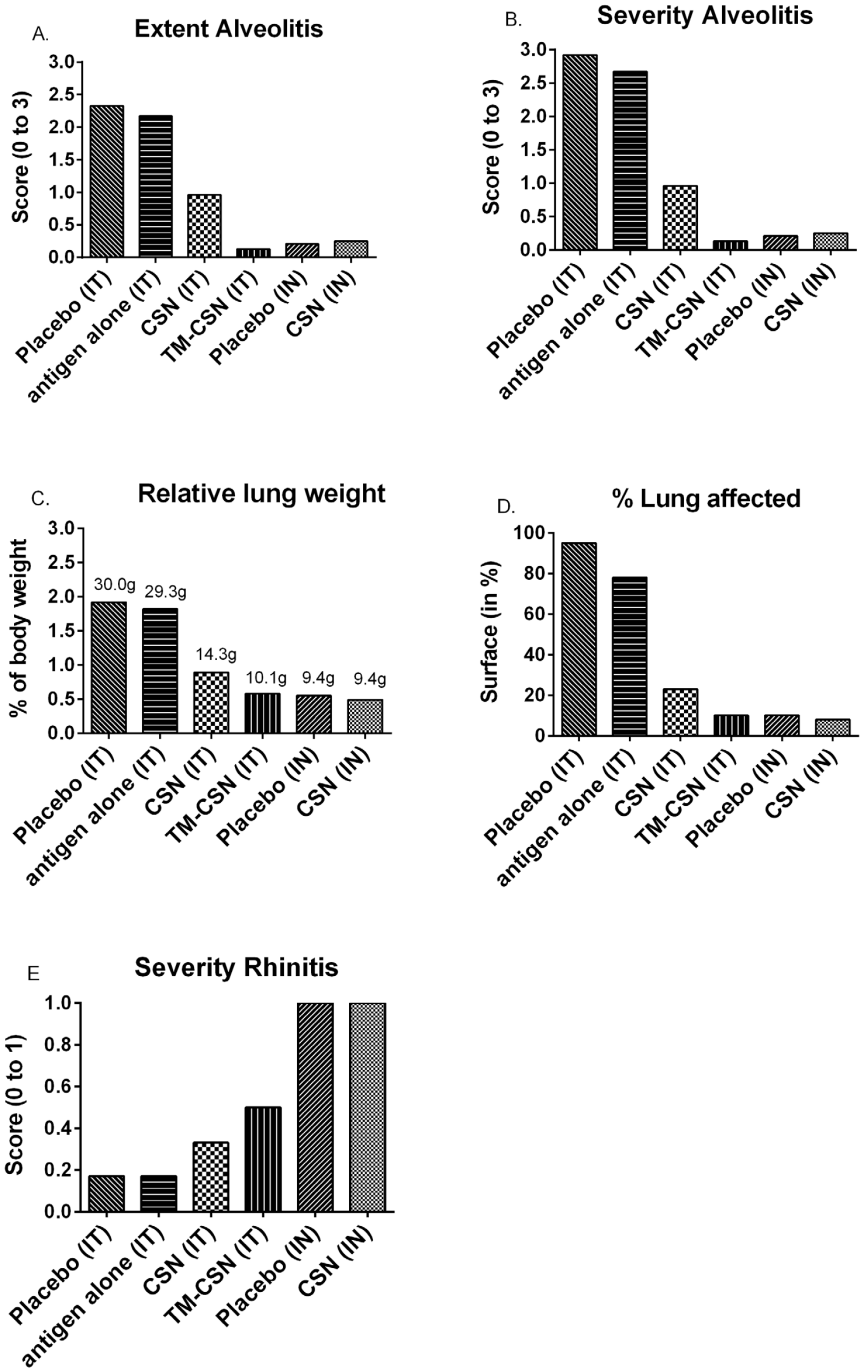
Histopathology in control and vaccinated ferrets post challenge. Histopathology was performed on ferrets that were euthanised according to schedule as well as animals euthanized prematurely on welfare groups and any decedents. In those ferrets that were not euthanised according to the schedule all had acute severe pneumonia or diffuse alveolar damage, which was attributed to the likely cause of death. None of those animals that were affected by encephalitis had to be euthanised prematurely. Each panel represents: (A) extent of alveolitis, (B) severity of alveolitis, (C) relative weight of lung, (D) percentage lung affected, and (E) severity of rhinitis.

Intratracheal challenge predominantly damaged the lung tissues (LRT), while intranasal challenge did not. In contrast intranasal challenge induced severe rhinitis (URT) in comparison to intratracheal challenge (Figure 5). Overall both TM-CSN and CSN adjuvanted vaccination of ferrets reduced LRT histopathological findings compared to placebo. Of those ferrets that died spontaneously or had to be euthanised prematurely all displayed acute severe pneumonia or diffuse alveolar damage, which was attributed to the challenge virus infection. None of those animals that were affected by encephalitis died spontaneously or had to be euthanised prematurely

## Discussion

We evaluated the immunogenicity and protective efficacy of ChiSys, a chitosan-based bioadhesive mucosal delivery system, as an intranasal vaccine adjuvant for an inactivated NIBRG-14 H5N1 subunit antigen. This is the first study to our knowledge in which a chitosan derivative has been used in a ferret Influenza challenge model.

The mechanism of action of chitosan and derivatives, as an adjuvant/delivery system for intranasally administered vaccine antigens, has not been fully elucidated. It is generally accepted that chitosan functions as a depot to protect and retain subcutaneously administered antigens at the local site [29] and it is probable that, as a mucoadhesive, chitosan plays a similar role in the nasal cavity by delaying mucociliary clearance of administered antigens. What is known is that solubility, degree of deacetylation, molecular weight, surface charge, and nature and degree of substitution are amongst the factors that will determine the effectiveness of the different chitosan forms as vaccine adjuvants. A glutamate salt form (CSN) and a trimethyl derivative of chitosan (TM-CSN) were evaluated in this study.

Vaccination twice with TM-CSN adjuvant (15 µg HA/dose) induced high serological titres against the vaccine antigen in 100% of animals with all serological assays, which completely protected the ferrets from lethal intratracheal challenge and provided sterilising immunity with no virus replication in URT, LRT, and CNS samples. Not only did TM-CSN vaccination induce high levels of antibodies, CSN adjuvanted vaccination also induced seroconversion and significantly reduced viral shedding and associated disease when ferrets were challenged by both the lethal intratracheal route as well as the intranasal route.

Serosurveillance studies in countries where H5N1 is endemic suggest that there are many people that have been exposed to H5N1 that have either had subclinical illness or have resolved their infection without the need to go to hospital [3]. In contrast the majority of patients seen in hospitals have presented with severe pneumonia, which had developed several days after symptom onset [30], [31]. While pneumonia is most frequently associated with H5N1 infected patients CNS involvement has also been observed [32].

A comparison of the route of H5N1 inoculation in ferrets and how it affects pathogenicity has shown that intranasal and intratracheal inoculation produce different disease profiles [33]. In the present study intratracheal challenge of placebo treated ferrets resulted in a lethal infection in the majority of animals and involved predominantly LRT disease with limited URT and CNS involvement. Intranasal challenge with the same total infectious titre of virus, although non-lethal, produced severe, predominantly URT disease with limited spread to the LRT and CNS. The design of this study included CSN adjuvanted intranasal vaccine challenged by both inoculation routes to test for protective efficacy against both disease outcomes. It would have been desirable to have challenged TM-CSN vaccine and antigen alone (unadjuvanted) vaccine groups by the intranasal route to evaluate these vaccines for a non-lethal challenge; however the study was restricted to 6 groups (36 animals) for ethical reasons.

An “ideal” influenza vaccine would induce long-term immunity that protects individuals from infection with the homologous virus, but would also generate cross-protective antibodies against heterologous strains of the same subtype. It would also allow for rapid manufacture in the event of a pandemic; require minimal viral antigen; remain stable at variable temperatures; and be safe, well-tolerated, and effective when given in a single dose [34]. In this study the TM-CSN vaccine induced strong cross-clade reactive antibody responses to H5N1 clades 2.1 and 2.2. The cross-clade reactive antibodies quantified post-vaccination suggest that vaccination with TM-CSN would likely protect against an H5N1 drifted variant challenge. To definitely establish the protective efficacy in an Influenza naïve ferret challenge model, the next step for this vaccine could be to challenge vaccinated animals with a different clade H5N1 virus. As demonstrated by Govorkova et al., H5N1-vaccinated ferrets that had had prior infection with epidemic influenza were still protected from challenge with an H5N1 virus that bore substantial antigenic differences from the vaccine antigen, even when serology assays did not detect cross-clade antibodies. [8]. Human subjects are expected to have had immunological exposure to Influenza within the first few years after birth [35]. As such it is thought that prior exposure to related, as well unrelated Influenza strains improves the response to subsequent antigenic exposure [36],[8]. It would be ideal if protective antibody responses were established after a single vaccination thereby reducing the period over which populations are potentially exposed to virus. Thus a single vaccination of ferrets with TM-CSN and CSN adjuvants followed by challenge could yield additional useful information.

Influenza H5N1 vaccines in ferrets have primarily been delivered by the intramuscular route [9],[10],[37],[38],[39] with fewer by the intranasal route [40],[41]. Many of these studies have demonstrated protection from a lethal challenge that was delivered either intranasally or intratracheally. Many vaccines have not shown both 100% seroconversion, cross clade seroprotection, as well as preventing virus replication in URT, LRT and CNS. In this study, we demonstrated protective immunity from lethal H5N1 Influenza infection in ferrets following intratracheal challenge. The TM-CSN adjuvanted vaccine conferred complete protection in ferrets with no disease or viral shedding in both the respiratory tract and the CNS. The CSN vaccinated ferrets were also protected from lethal infection and had reduced viral replication, clinical signs, and pathological findings.

## Supporting Information

File S1 **Supporting information for animal husbandry, virus neutralisation methods, statistics, and detailed serology tables.** Table S1. Group serological responses after 1 and 2 vaccinations (HAI, VN, and SRH) to homologous A/Vietnam/1194/2004 [H5N1] - clade 1. Table S2. Group serological responses after 1 and 2 vaccinations (HAI, VN, and SRH) to heterologous A/Indonesia/05/2005 [H5N1] -clade 2.1. Table S3. Group serological responses after 1 and 2 vaccinations (HAI, VN, and SRH) to heterologous A/Turkey/Turkey/1/2005 [H5N1] - clade 2.2.
(DOCX)
