# Supplementary material for: Intranasal H5N1 Vaccines, Adjuvanted with Chitosan Derivatives, Protect Ferrets against Highly Pathogenic Influenza Intranasal and Intratracheal Challenge
Source: PLoS One. 2014 May 21;9(5):e93761. doi: 10.1371/journal.pone.0093761 (PMC4029577; doi:10.1371/journal.pone.0093761)
Supplement: File S1 — Supporting information for animal husbandry, virus neutralisation methods, statistics, and detailed serology tables. Table S1. Group serological responses after 1 and 2 vaccinations (HAI, VN, and SRH) to homologous A/Vietnam/1194/2004 [H5N1] - clade 1. Table S2. Group serological responses after 1 and 2 vaccinations (HAI, VN, and SRH) to heterologous A/Indonesia/05/2005 [H5N1] -clade 2.1. Table S3. Group serological responses after 1 and 2 vaccinations (HAI, VN, and SRH) to heterologous A/Turkey/Turkey/1/2005 [H5N1] - clade 2.2. (DOCX) [file pone.0093761.s001.docx]

**Supporting information:**

**Animal husbandry**

Animals were maintained in standard housing during acclimatisation and vaccination phase, and provided with commercial food pellets and water *ab libitum*. The challenge phase was performed in negatively pressurised isolator cages at animal biosafety level 3^+^ (BSL3^+^) conditions. Animal welfare was observed on a daily basis (more frequently after inoculation) and any animal which gave cause for concern on welfare grounds was closely observed. Any animal which showed signs of moderate/significant deterioration post inoculation were euthanized to prevent any suffering. Animal procedures including immunisation, collection of blood and nasal/throat swabs were performed under light anesthesia using Ketamine (intramuscular 4-8 mg/Kg). Viral inoculation and implantation of temperature transponders as well as euthanasia by exsanguinations were performed under anaesthesia with a mixture of Ketamine (intramuscular 4-8 mg/Kg) and medetomidine (intramuscular 0.1 mg/Kg). Atipamezole (intramuscular 0.5 mg/Kg) was administered to reverse the effects of medetomidine.

**Virus neutralisation methodology**

Serum samples were heat inactivated at 56°C for 30 minutes and then serial twofold dilutions were performed starting from 10^-1^ dilution. The diluted samples were incubated at 37°C for 1 hour with 100TCID_50_. The mixture of sample-virus (100 µl/well) was added to the MDCK-SIAT1 cell layer (90% confluent) and then incubated at 37°C / 0.5%CO_2_ for 5 days. At the end of the incubation period the cytopathogenic effect (CPE) was checked to calculate the neutralisation titre (NT). The NT was defined as the serum dilution for which 50% of the cultures were protected from a virus-induced cytopathogenic effect

**Statistical Analysis**

**Viral titres:** Virus titration data for nasal and throat swabs was recorded daily from Day 49 through to Day 54. The Area under the curve for the viral data was calculated and the AUC analyzed using ANOVA. The estimated treatment differences for each treatment comparison were obtained for each treatment comparison.

Viral load on the Day of death were analyzed using ANOVA. Values that were below the level of quantification (LQ) were replaced with 0.5*LQ for summary and analysis. Estimates of the treatment median differences were obtained from the analysis. Comparisons of mean viral titres in lungs, nasal turbinates, brain and olfactory bulb at death were analysed by the Kruskal‑Wallis test.

**% Body Weight change analysis:** body weight change was analysed using a repeated measures analysis of covariance in which the baseline weight was used as a covariate, with fixed effects for day and group and random effects for animal. The area under the curve (AUC) obtained from the plot of body weight versus dpi.

**Peak Body Temperature change ^0^C:** The peak (maximum) body temperature recorded after 00:00 hours on Day 49 was identified and the maximum change from baseline calculated. The mean temperature recorded between 00:00 hours on Day 45 and 23:50 hours on Day 48 (i.e. prior to challenge) was calculated and used to denote the baseline temperature for each animal. The maximum increase (peak) in body temperature from baseline was analysed using the Kruskal‑Wallis test.

**RESULTS**

**Table S1. Serological responses of immmunised or placebo ferrets to homologous A/Vietnam/1194/2004 [H5N1]**

|  | Anti-A/ Vietnam/1194/2004 (Clade 1) serum antibody titre (range) | | | | | | | |
| --- | --- | --- | --- | --- | --- | --- | --- | --- |
|  | HAI (Turkey blood) | | HAI (Horse blood) | | VN | | SRH | |
| Intranasal Vaccination (15μg) | GMT | Seroconverted^a^ | GMT | Seroconverted^a^ | GMT | Seroconverted^a^ | GMT | Seroprotected^c^ |
| Unadjuvanted / Antigen alone |  |  |  |  |  |  |  |  |
| 1 vaccination Day 21 | <5 | 0/6 | <10 | 0/6 | <5 | 0/6 | <4 | 0/6 |
| 2 vaccinations Day 42 or 48^d^ | <5 | 0/6 | <10 | 0/6 | <5 | 0/6 | <4 | 0/6 |
| CSN Adjuvanted |  |  |  |  |  |  |  |  |
| 1 vaccination Day 21 | <5 (<5-40) | 2/12 | 7 (<10-40) | 0/12 | 6 (<5-14) | 0/12 | 5 (<4-20) | 0/12 |
| 2 vaccinations Day 42 or 48 ^d^ | 22 (<5-240) | **7/12 ^*^** | 34 (<10-320) | **5/12 ^*^** | 34 (5-113) | **8/12** **^*^** | 23 (<4-71) | **7/12 ^*^** |
| TM-CSN Adjuvanted |  |  |  |  |  |  |  |  |
| 1 vaccination Day 21 | 10 (<5-60) | **3/6 ^*^** | <10 | 0/6 | <5 | 0/6 | <4 | 0/6 |
| 2 vaccinations Day 42 or 48 ^d^ | 259 (160-640) | **6/6 ^*^** | 403 (160-1280) | **6/6 ^*^** | 90 (57-226) | **6/6 ^*^** | 58 (28-95) | **6/6 ^*^** |
| Placebo |  |  |  |  |  |  |  |  |
| 2 vaccinations Day 42 or 48 ^d^ | <5 | 0/12 | <10 | 0/12 | <5 | 0/12 | 6 (<4-33) | 1/12 |

Values in bold and with an * - represent significant differences compared to placebo; p<0.05 by Fisher’s exact test, two tailed

^a^ HAI seroconverted proportion includes those ferrets that had a ≥4 fold rise from baseline / total number of ferrets

^b^ VN seroconverted proportion includes those ferrets that had a ≥4 fold rise from baseline / total number of ferrets

^c^ SRH seroprotected proportion includes those ferrets that had a ≥25mm^2^ haemolysis area / total number of ferrets

^d^ For serology post 2^nd^ vaccination the highest value achieved on either Day 42 or 48 was used

**Table S2. Serological responses of immunized or placebo ferrets to A/Indonesia/05/2005 [H5N1]**

|  | Anti-A/ Indonesia/05/2005 (Clade 2.1) serum antibody titre (range) | | | | | | | |
| --- | --- | --- | --- | --- | --- | --- | --- | --- |
|  | HAI (Turkey blood) | | HAI (Horse blood) | | VN | | SRH | |
| Intranasal Vaccination (15μg) | GMT | Seroconverted^a^ | GMT | Seroconverted^a^ | GMT | Seroconverted^a^ | GMT | Seroprotected^c^ |
| Unadjuvanted / Antigen alone |  |  |  |  |  |  |  |  |
| 1 vaccination Day 21 | <10 | 0/6 | <10 | 0/6 | <5 | 0/6 | 4 (<4-7) | 0/6 |
| 2 vaccinations Day 42 or 48 ^d^ | <10 | 0/6 | <10 | 0/6 | <5 | 0/6 | 4 (<4-7) | 0/6 |
| CSN Adjuvanted |  |  |  |  |  |  |  |  |
| 1 vaccination Day 21 | <10 | 0/12 | <10 (<10-40) | 1/12 | <10 | 0/12 | 4 (<4-10) | 0/12 |
| 2 vaccinations Day 42 or 48 ^d^ | 10 (<10-40) | 4/12 | 12 (<10-80) | 2/12 | 8 (<5-28) | 3/12 | 9 (<4-38) | 3/12 |
| TM-CSN Adjuvanted |  |  |  |  |  |  |  |  |
| 1 vaccination Day 21 | <10 | 0/6 | <10 (<10-10) | 0/6 | <5 | 0/6 | <4 | 0/6 |
| 2 vaccinations Day 42 or 48 ^d^ | 40 (<10-160) | **5/6** **^*^** | 101 (<10-640) | **5/6 ^*^** | 19 (<5-28) | **4/6 ^*^** | 21 (<4-79) | **4/6 ^*^** |
| Placebo |  |  |  |  |  |  |  |  |
| 2 vaccinations Day 42 or 48 ^d^ | <10 | 0/12 | <10 | 0/12 | <5 (<5-10) | 0/12 | <4 (<4-10) | 0/12 |

Values in bold and with an * - represent significant differences compared to placebo; p<0.05 by Fisher’s exact test, two tailed

^a^ HAI seroconverted proportion includes those ferrets that had a ≥4 fold rise from baseline / total number of ferrets

^b^ VN seroconverted proportion includes those ferrets that had a ≥4 fold rise from baseline / total number of ferrets

^c^ SRH seroprotected proportion includes those ferrets that had a ≥25mm^2^ haemolysis area / total number of ferrets

^d^ For serology post 2^nd^ vaccination the highest value achieved on either Day 42 or 48 was used

**Table S3. Serological responses of immunized or placebo ferrets to A/Turkey/Turkey/1/2005 [H5N1]**

|  | Anti-A/ Turkey/Turkey/1/2005 (Clade 2.2) serum antibody titre (range) | | | | | | | |
| --- | --- | --- | --- | --- | --- | --- | --- | --- |
|  | HAI (Turkey blood) | | HAI (Horse blood) | | VN | | SRH | |
| Intranasal Vaccination (15μg) | GMT | Seroconverted^a^ | GMT | Seroconverted^a^ | GMT | Seroconverted^a^ | GMT | Seroprotected^c^ |
| Unadjuvanted / Antigen alone |  |  |  |  |  |  |  |  |
| 1 vaccination Day 21 | <10 | 0/6 | <10 | 0/6 | <10 | 0/6 | 5 (<4-10) | 0/6 |
| 2 vaccinations Day 42 or 48 ^d^ | <10 | 0/6 | <10 | 0/6 | <10 | 0/6 | 5 (<4-13) | 0/6 |
| CSN Adjuvanted |  |  |  |  |  |  |  |  |
| 1 vaccination Day 21 | <10 | 0/12 | <10 (<10-40) | 2/12 | <10 | 0/12 | 5 (<4-7) | 0/12 |
| 2 vaccinations Day 42 or 48 ^d^ | <10 (<10-20) | 2/12 | <10 (<10-80) | 2/12 | <10 (<10-56) | 2/12 | 17 (<4-44) | 6/12 |
| TM-CSN Adjuvanted |  |  |  |  |  |  |  |  |
| 1 vaccination Day 21 | <10 | 0/6 | <10 (<10-20) | 0/6 | <10 | 0/6 | <4 | 0/6 |
| 2 vaccinations Day 42 or 48 ^d^ | 18 (<10-80) | **3/6 ^*^** | 25 (<10-640) | 2/6 | 50 (28-113) | **6/6 ^*^** | 44 (28-79) | **6/6 ^*^** |
| Placebo |  |  |  |  |  |  |  |  |
| 2 vaccinations Day 42 or 48 ^d^ | <10 | 0/12 | <10 | 0/12 | <10 | 0/12 | 7 (<4-38) | 2/12 |

Values in bold and with an * - represent significant differences compared to placebo; p<0.05 by Fisher’s exact test, two tailed

^a^ HAI seroconverted proportion includes those ferrets that had a ≥4 fold rise from baseline / total number of ferrets

^b^ VN seroconverted proportion includes those ferrets that had a ≥4 fold rise from baseline / total number of ferrets

^c^ SRH seroprotected proportion includes those ferrets that had a ≥25mm^2^ haemolysis area / total number of ferrets

^d^ For serology post 2^nd^ vaccination the highest value achieved on either Day 42 or 48 was used
